# Central Projection of Pain Arising from Delayed Onset Muscle Soreness (DOMS) in Human Subjects

**DOI:** 10.1371/journal.pone.0047230

**Published:** 2012-10-08

**Authors:** Katharina Zimmermann, Caroline Leidl, Miriam Kaschka, Richard W. Carr, Pavel Terekhin, Hermann O. Handwerker, Clemens Forster

**Affiliations:** Department of Physiology and Pathophysiology, Friedrich-Alexander-University Erlangen-Nuremberg, Erlangen, Germany; University of Minnesota, United States of America

## Abstract

Delayed onset muscle soreness (DOMS) is a subacute pain state arising 24–48 hours after a bout of unaccustomed eccentric muscle contractions. Functional magnetic resonance imaging (fMRI) was used to examine the patterns of cortical activation arising during DOMS-related pain in the quadriceps muscle of healthy volunteers evoked by either voluntary contraction or physical stimulation. The painful movement or physical stimulation of the DOMS-affected thigh disclosed widespread activation in the primary somatosensory and motor (S1, M1) cortices, stretching far beyond the corresponding areas somatotopically related to contraction or physical stimulation of the thigh; activation also included a large area within the cingulate cortex encompassing posteroanterior regions and the cingulate motor area. Pain-related activations were also found in premotor (M2) areas, bilateral in the insular cortex and the thalamic nuclei. In contrast, movement of a DOMS-affected limb led also to activation in the ipsilateral anterior cerebellum, while DOMS-related pain evoked by physical stimulation devoid of limb movement did not.

## Introduction

Functional magnetic resonance imaging (fMRI) has revealed several aspects on how acute and chronic pain is processed in the human brain. Most pain imaging studies predominantly focus on the investigation of cerebral processing of cutaneous pain in spite of the fact that muscle and/or musculoskeletal pain entail more common clinical problems and are also widespread conditions amongst athletes [1]. Apparently, sensory and motor characteristics distinguish cutaneous pain from muscle pain and activate different brain areas [2]. A series of studies has addressed psychophysical [3]–[5], peripheral [4], and spinal [6] mechanisms of experimentally induced muscle pain. These and other studies used the application of exogenous stimuli as models to evoke phasic or tonic muscle pain sensations. Electrical stimulation is used as a nonspecific method to induce pain by direct activation of nerve and muscle fibers. However, high currents are required to activate thinly and non-myelinated nociceptive fibers. Consequently, evoking pain results in unwanted activation of non-nociceptive nerve and muscle fibers and muscle twitches and limb movement may accompany the stimulation and adulterate functional brain imaging [7], [8]. Another frequently used model consists in the injection of chemicals into the muscle tissue, such as hypertonic saline, acid phosphate buffer, bradykinin or glutamate; these substances induce cramp like diffuse pain mimicking the chronic pain observed by patients. However, referred pain, superficial mechanical hyperalgesia and autonomic reactions also occur and influence brain imaging studies [9]–[12]. Electrical stimulation, injection of hypertonic saline or acid phosphate buffer have been used in conjunction with event-related functional magnetic resonance imaging (fMRI) and positron emission tomography (PET) to study the supraspinal processing of muscle pain [2], [13]–[17].

To study the central processing of muscle pain by fMRI in a more physiological context we used eccentric muscle exercise and subsequent painful delayed onset muscle soreness (DOMS). In unaccustomed subjects eccentric exercise frequently results in the formation of DOMS, a form of subacute muscle pain, which appears 24 to 72 hours after exercise. Clinically, DOMS is a common but self-limiting condition that requires no treatment. In the present study, we induced DOMS locally confined to the right-sided quadriceps muscle group. Our study is the first to use DOMS to study its pain-related cerebral activation pattern in an fMRI environment. Within the fMRI environment, we evoked DOMS-related pain by voluntary painful contraction, but also by externally applied physical stimulation in the form of localized pressure which was moved over the sore partitions of the muscle by using a custom-designed heavy marble roll. The latter paradigm allowed separation of DOMS-related pain from limb movement-related brain activation. Our study is the first to highlight the central pathophysiology and activation pattern of this very common form of muscle pain.

## Materials and Methods

### Subjects

The fMRI study included eight right-handed healthy volunteers (5 males, 3 females, mean age 26.2 years, range 20–34 years), who were either students or physicians and already experienced in participating in psychophysical studies. All participants participated voluntarily in the experiments and none of them were taking medication that might have interfered with the induction of subacute muscle soreness or with pain sensations (i.e. analgesics, anesthetics or other centrally acting drugs). The study adhered to the claims of the Declaration of Helsinki and the protocol was approved by the local ethics committee (Institutional Review Board of the University Erlangen-Nuremberg).

### Induction of delayed onset muscle soreness (DOMS)

DOMS is a physiological pain state that occurs following a bout of eccentric muscle contractions and peaks in intensity 24-48 hours after exercise [18]. To induce DOMS in the quadriceps muscle group, subjects performed 120–150 up-down step cycles with their right leg. A step cycle consisted of stepping up onto a small platform with the right leg (concentric quadriceps contraction) then stepping back down with the left leg which forces the right leg to bear the body weight (eccentric quadriceps contraction). The height of the platform was adjusted such that the included knee angle was 90° or less when the right foot was placed on the platform, i.e. the eccentric contraction was performed over a long quadriceps muscle length. 36 to 48 hours later, subjects were psychophysically evaluated. Eight of the eleven subjects experienced intense soreness and long lasting pain in their quadriceps muscle upon contraction or physical stimulation and were admitted to the fMRI experiment. None of the subjects reported resting pain.

### Experimental pain model and intensity rating

The intensity of the pain was quantified and monitored using the visual analogue scale (VAS) ranging from 0 to 100% as previously described [19]. The rating scale was fed back to the subject as a light bar representing the levels 0–100% of the common VAS scale, whereby “0%” signifies no pain, “100%” marks maximal imaginable pain. For psychophysical measurements, the subjects sat reclined in a dental chair and the paradigm was carried out as later in the fMRI sessions, with the only difference that subjects were asked to operate the VAS with a turning knob to rate the pain as perceived during the contractions and during stimulation with the heavy marble roll. As for the contraction task, the subjects were instructed to perform a maximal isometric contraction of their painful and non-painful quadriceps muscle, but also to refrain from movements of the head as much as possible. Only those subjects who judged the contraction/stimulation with the roll on the sore side as painful, and attributed a rating of at least 15% on average were admitted to the fMRI experiment. The success rate (of achieving painful DOMS with this training method) exceeded 70%, i.e. eight of the eleven subjects that were initially trained by eccentric exercise, had developed painful soreness and were admitted to the scanner to record their brain activation in response to stimulation of the painful muscles. For control purposes, the subjects were asked after the fMRI experiment, to estimate the intensity of pain they had experienced during both self-controlled contraction and physical stimulation. In all subjects the pain ratings confirmed the ratings that were obtained during the psychophysical training session prior to the fMRI measurement.

### FMRI stimulation procedures

**Figure 1:**
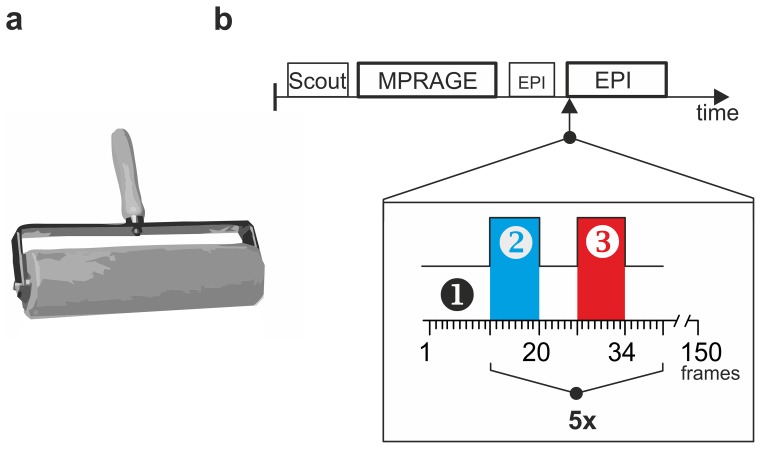
Stimulus conditions and experimental paradigm. **a.** Muscle pain was evoked in a muscle group suffering DOMS during an fMRI measurement by two paradigms: voluntary contraction of the painful muscle (paradigm 1) and painful physical stimulation of the sore muscles with a 1.75 kg heavy marble rolling pin (paradigm 2). **b.** The scanning procedure included 5 repetitions of the same stimulus combination (5x EPI). A Scout epoque - used to determine the position of the subject's head within the scanner- is followed by the acquisition of the high-resolution anatomical MPRAGE data set. A short EPI sequence is required to accustom the subject to the unfamiliar noise of the following EPI scans. The numbers in the block design (x-axis) are equivalent to scanning frames and one frame corresponds to 3 s acquisition time. The conditions were as follows: 1: resting condition (baseline); 2: contralateral painless quadriceps contraction or painless physical stimulation; 3: ipsilateral painful quadriceps contraction or painful physical stimulation.

Shortly before subjects attended the fMRI scanner they were instructed to refrain from movement as much as possible. In the scanner the subjects' head was immobilized by fixing the head binaurally with a thrust device. A mirror was mounted above the eyes to allow the subject to see the light indicating the protocol, which was positioned outside the scanner. Two paradigms were used to evoke DOMS-associated muscle pain in the fMRI environment. The first paradigm involved alternate repeated active contractions of the right (sore) and the left (sound) quadriceps muscle group and was phasic in nature. The stimulus is identical to an isometric contraction and the evoked pain proves to be suited for the requirements of a functional MRI stimulus design that calls for stimuli with fast on- and offset. The whole paradigm consisted of 5 cycles of alternating non-painful (control condition) and painful contractions (stimulus) with eleven interleaved resting conditions (baselines), respectively 24 and 18 seconds **(** **Fig. 1** **)**. A red light indicated the stimulation period, during which the subjects were asked to repeatedly contract their right (sore) quadriceps muscle. Indicating control condition, the light switched to green, and the subjects to their healthy, left quadriceps group, maintaining phasic contractions over the period of the non-painful stimulus (control condition). In detail, for the contraction-related task, the subjects were advised to contract their quadriceps muscle either with maximum strength or until maximal tolerable pain was perceived and then to relax and contract the muscle again. One cycle of contraction and subsequent relaxation resulted in approximately 3 seconds and was repeated during the 24 seconds of stimulation until the light switched off to indicate baseline condition. The second paradigm consisted in physical stimulation of the sore areas of the thigh by externally applied localized pressure. It was used to separate brain activation in response to deep muscle pain from activation resulting from muscle contraction. Due to the slow adaptation properties of sensitized nociceptors (in deep tissues), pain sensation in response to tonic locally applied pressure dampens within seconds [20], [21]. In order to obtain a persistent and constant pain sensation during the 24 seconds of stimulation, the pressure was moved constantly within the sore target area of the quadriceps. This was achieved by using a custom-designed marble roll. The marble roll was fixed in a brass handle and did not interfere with the signal of the scanner (**Fig. 1a**). During the 24 s of stimulation, the experimenter gently rolled the device back and forth over the sore (or contralateral control) target areas. This procedure is referred to as “physical stimulation”. To evoke comparable pain rating and achieve constant stimulation conditions across the group of subjects, the most painful areas of the thigh were marked with tape in each subject prior to the fMRI measurement (tape was placed and stimulation performed through the trousers in order to prevent activation of brain areas due to stimulation of cutaneous cold receptors by the marble roll). Accordingly, the marble roll was held perpendicularly to the surface and rolled only over the marked areas. Furthermore, care was taken to exert pressure only by imposing the 1.75 kg dead load of the roll and no additional force. This way of stimulation produced in all subjects a deep and constant painful feeling with fast on- and offset. Few hours before accessing the fMRI scanner, each subject underwent a training session in order to make the subjects familiar with the stimulation procedure, but also to evaluate intensity and time course of the pain and to find and mark the most painful areas of the thigh with tape.

### Image acquisition, study design and MRI sequence order

Images were collected with a 1.5 Tesla MRI scanner (Magnetom Sonata, Siemens, Erlangen, Germany) using a standard quadrature head coil. Preceding the functional measurements, a high resolution data set of each subject’s brain was acquired using a T1-weighted, 3D gradient-echo pulse sequence (MPRAGE: magnetization prepared rapid acquisition gradient echo; voxel size  = 1,0 mm^3^; TR/TE  = 1950 ms/4.38 ms, FOV  = 256 mm, 256×256 matrix, 160 slices, FOV  = 256 mm, slice thickness  = 1 mm). Functional scans were acquired using a blood-oxygen-level-dependent (BOLD) protocol with a T2-weighted gradient echo-planar imaging (EPI) sequence (TR/TE/θ  = 3000 ms/60 ms/90°; slice thickness  = 4 mm; interslice interval  = 1 mm, FOV  = 220 mm, 64×64 matrix). 20 axial slices were placed such that the best possible whole brain coverage was obtained, usually from the top of the cortex to the base of the cerebellum. The first three images were discarded to account for spin saturation effects thus eliminating non-equilibrium effects of the magnetization. MRI sequences were measured in the following order: anatomical scout, MPRAGE, and two EPI sequences. The first EPI was short and applied to accustom the subject to the noise. The second EPI consisted of 150 whole brain acquisitions divided into 5 cycles and entailing the protocol as described above. Eight sequences were acquired during the stimulus period time (24 s) and six during the interposed baseline periods (resting condition, 18 s; see **Fig.** **1**).

### Data processing and statistical analysis

Imaging data analysis, registration, visualisation and statistical analyses were performed with the BrainVoyager QX software package (Brain Innovation B.V., Maastricht; The Netherlands). Pre-processing of the data was performed as previously described [22] and included three-dimensional motion correction, temporal Gaussian smoothing of 4 s, spatial Gaussian smoothing of 4 mm, linear detrending and temporal high pass filtering using 0.01 Hz. The 3D-MPRAGE data set was transformed into the standard stereotactic space [23] and the T2 weighted images were realigned to this high-resolution 3D-data set [24] thus enabling a 3D-reconstruction of the activation maps. Fixed-effect analyses were performed and to search for clusters of activation, we used a block design with two conditions (stimulus vs. control) and an interposed baseline (see **Fig.** **1b**). The stimulus sequence pattern was convoluted with a hemodynamic response function to account for the expected delay and devolution of the BOLD signal [25], [26] and served as a basis for the calculation of the correlation coefficients of the respective cluster. Blocks of contraction activity within the stimulation pattern served as independent predictors for a general linear model (GLM). Activation was detected by correlating the time course of the BOLD-signal of each voxel with the predictor pattern of the GLM. Contrasts of interest were calculated by subtraction analysis and t-tests were calculated as differential responses, resulting in statistical maps (painful vs. non-painful states [contraction-/ physical stimulation-induced muscle pain vs. contralateral control]). P-values <5*10^−6^ (uncorrected) are marked in Table 1. The subtraction analyses were complemented with separate evaluation of the activation pattern of each stimulus versus its control. Analyses were performed at the group level (multi-study). Location of activated clusters was verified using a printed atlas [27] and labelled using Tailarach's nomenclature [23] by means of the Tailarach Daemon (Research Imaging Center, University of Texas Health Center, San Antonio, TX, USA). Table 1 lists clusters with a minimum number of 250 voxels corresponding to 250 mm^3^. We focussed on clusters located in the following brain areas: frontal and parietal lobe, insula, cingulum, thalamus, basal ganglia and the cerebellum. Processing of the clusters included identification of Brodman's Area (BA; or closest BA in case the centre of the cluster was located in the white matter), determination of spatial centre (coordinates), and their statistical significance (t-value). Psychophysical data were recorded with custom made software.

**Table 1:** Brain regions with increased activity during DOMS: comparison between painful voluntary contraction and physical stimulation with a rolling pin.

	voluntary contraction						physical stimulation				
			T values						T values		
	BA	x, y, z	contrast	Pain	Con		BA	x, y, z	contrast	Pain	Con
**Right Brain**											
**Parietal Lobe**											
Postcent. Gyrus (S1)	**5**	9, −45, 63	−5.3*	5.8*	11.5*		**5/7**	9, −42, 66	−9.7*	−3.5	7.1*
Precuneus (SSA)	**7**	9, −52, 62	−3.2	3.4	6.9*		**7**	11, −54, 61	−8.6*	−8.5*	0.8
**Frontal Lobe**											
Precentral Gyrus (M1)	**4**	7, −36, 62	−4.4*	7.0*	11.8*		**4**	17, −24, 65	−4.6	−5,8*	−0.9
Paracentr. Lob.(M1)	**6**	6, −33, 62	−4.4	7.0*	11.7*		**6**	7, −40, 69	−8.8*	−3.3	6.1*
Midd. Front. Gyrus							**8**	26, 33, 36	7.8*	4.2	−4.2
**Insula**											
Anterior	**13**	31,14,17	5.4	8.4*	2.6		**13**	38, 8, 11	7.4*	10.0*	2.1
Medial											
Posterior							**13**	31, −26, 21	−8.2*	−1.5^0.1^	7.4*
**Cingulum**											
post. ACC	**24**	5, 9, 37	5.4*	8.7*	2.9		**32**	9, 18, 33	7.0*	5.0*	−2.5
PCC	**31**	3, −31, 28	4.7*	7.8*	2.7		**31**	5, −31, 30	4.5	2.2	−2.6
**Thalamus/Basal Ganglia**											
Medial Dorsal Nucl.		3, −12, 5	4.8*	8.3*	3.2			10, −14, 10	5.1*	3.1	−2.4
Lentiform Nucleus								20, −2, 8	6.8*	8.6*	1.3
**Cerebellum**											
Anterior Lobe		12, −40, −23	10.5*	15.3*	3.9			16, −38, −23	7.7*	6.7*	−1.6
Ant. Lobe, Culmen		33, −53, −22	6.5*	13.2*	6.2*						
Ant. Lobe, Culmen		3, −50, −13	10.3*	17.2*	6.1*						
Post. Lobe, Declive		18, −69, −21	6.3*	12.4*	5.5*						
**Left Brain**											
Postcentral Gyrus (S1)	**3**	−12, −40, 67	20.7*	22.1*	−0.3		**3**	−12, −40, 67	17.3*	14.0*	−4.7
Precuneus (SSA)	**7**	−13, −53, 55	7.1*	9.1*	1.5		**7**	−11, −43, 55	10.0*	5.2*	−5.5*
IPL /S2	**40**	−39, −31, 26	6.8*	10.0*	2.7		**40**	−51, −30, 23	6.0*	13.9*	6.3*
SPL							**7**	−18, −45, 62	13.1*	12.7*	−1.5^1^
**Frontal Lobe**											
Precentral Gyrus	**4–5**	−10, −29, 66	15.0*	20.1*	3.9		**4–5**	−10, −29, 66	15.0*	11.0*	-5.3
Paracentr. Lob. (M1)							**6**	−8, −31, 61	14.9*	14.4*	−5.7
Med.F.Gyrus (SMA)	**6**	−5, −21, 64	9.6*	19.1*	8.6*		**6**	−10, −29, 70	15.0*	11.4*	−4.7*
Midd. F. Gyrus							**46**	−44, 38, 26	6.3*	7.2*	0.4
**Temporal Lobe**											
STG	**41**	−48, −30, 11	4.4*	8.0*	3.2		**22**	−51, 4, 5	8.3*	9.8*	−0.8
**Insula/Claustrum**											
Anterior	**13**	−35, 10, 16	4.1	10.3*	5.9*		**13**	−36, 16, −1	7.5*	7.6*	−0.5
Medial	**13**	−30, −8, 16	7.2*	15.4*	7.6*		**13**	−35, 0, 10	10.0*	14.1*	−3.2
Posterior	**13**	−32, −23, 13	8.3*	12.5*	3.5		**13**	−35, −24, 15	10.1	11.8*	0.8
**Cingulum**											
CMA	**24/31**	−11, −22, 41	5.5*	7.8*	1.9	**24/31**		−9, −11, 43	7.9*	6.2*	−2.3
PCC	**23**	−10, −31, 28	5.7*	10.2*	4.0	**23**		−7, −33 28	5.3*	4.4	−1.3
**Thalamus/Basal Ganglia**											
Ventral Nuclei		−19, −23, 5	6.5*	10.1*	3.0			−13, −23, 10	9.1*	5.1*	−4.8*

p-values <0.000005 are indicated as “*” (uncorrected); S1: primary somatosensory cortex, M1: primary motor cortex; S/IPL: superior/inferior parietal lobule; STG: superior temporal gyrus; SSA: somatosensory association cortex; SMA: supplementary motor area; ACC: anterior cingulate cortex; PCC: posterior cingulate cortex; CMA: cingulate motor area; BA: Brodmann Areas or closest BA in vicinity; “Pain”, T-values are indicated for Pain = painful condition and Con = control condition and contrast; x, y, z: Talairach coordinates.

## Results

### Psychophysical ratings and painfulness of the stimulus

**Figure 2:**
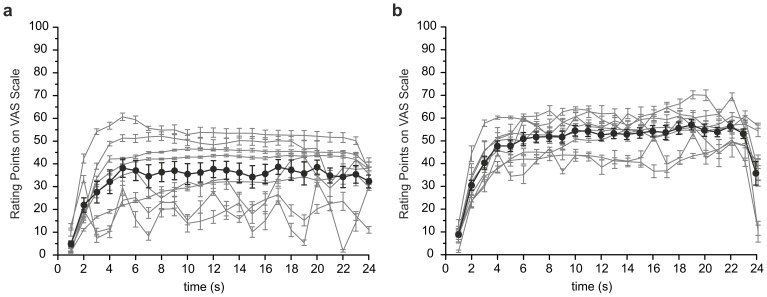
Painfulness of the stimulus. To evaluate the painfulness of DOMS all subjects rated the pain perceived during contraction or physical stimulation. Subjects contracted the thigh repeatedly during 5 cycles of 24 seconds each or the respective muscle was stimulated with the heavy marble rolling pin by the experimenter (compare protocol in **Fig.** **1**). Individual pain-ratings (VAS-scale) are given as averages across the 5 cycles (grey lines) and as common average (black circles) across the group. On average the subjects rated the painfulness of **(a)** the voluntary contraction to 35±14 % and **(b)** the physical stimulation as 50±8 % on the VAS scale. Thus, physical stimulation was judged as more painful (n = 8 each; p = 0.02 Mann-Whitney U-test; error bars show S.E.M.; grey areas indicate averaged highs and lows). Subjects had no resting pain and experienced pain only during stimulation of the affected limb and not of the control side.

To quantify the pain, all subjects had to rate the pain perceived during contraction and physical stimulation of the sore quadriceps before the fMRI measurement. For this purpose the subjects were instructed to contract the painful muscles in 5 repeated cycles of 24 seconds each (compare protocol in **Fig.** **1b**). The rating of the pain intensity during these 5 cycles was then averaged for each subject and is depicted for paradigm 1 (contraction) in **Fig.** **2a** and for paradigm 2 (physical stimulation) in **Fig.** **2b**. Intra-individual variance was low for the 5 cycles of stimulation. The subjects rated the pain of the contraction on average to 35±8 % on the VAS scale (n = 8), reaching maximum values of up to 61%. Physical stimulation was rated more painful, and was judged on average to 50±8% with maximum values of up to 70 % on the VAS scale. While all subjects judged the time course of the pain during physical stimulation as constant and plateau-like; three subjects described alternating increasing and decreasing pain during the contraction task (compare grey lines in **Fig.** **2**). We assume that the fluctuations in the pain sensation in these particular subjects likely reflect increasing pain during contraction and partial relief during relaxation.

### Pain-related BOLD-signals in cortical areas

**Figure 3:**
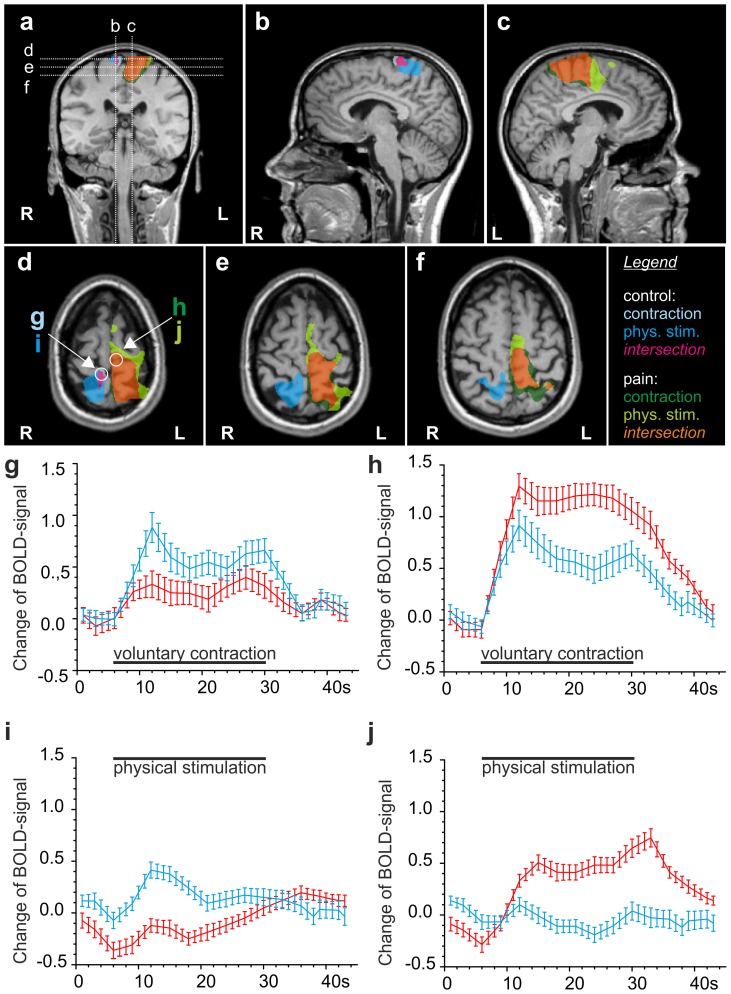
Comparison of DOMS-related activation by contraction and physical stimulation in M1 and S1. Multi studies of activations by both stimuli in a standardized Talairach brain shown as activation maps **(a–f)** and as increases in hemodynamic response function **(g–j)**. Clusters were calculated by GLM contrasts between painful and non-painful stimulation conditions (t≥5). Legend: Light blue: activation by painless contraction; dark blue: activation by painless physical stimulation; magenta: common activation by both painless stimuli; dark green: activation by painful contraction; light green: activation by painful physical stimulation; orange: common activation by both painful stimuli. **(a)** coronal section showing orientation of sagittal sections b and c and transverse sections d, e and f. **(b, c)** Sagittal sections of the parasagittal cortical areas of both hemispheres illustrating the area somatotopically related to the respective thigh (M1 and S1). Painful stimulation –contraction as well as physical stimulation- activate areas expanding into the cingulate cortex. **(d, e, f)** Painless physical stimulation is centred in postcentral gyrus S1. Both contraction and physical stimulation activate precentral M1. Activation in response to painful stimulation expands over large areas of M1 and S1 with physical stimulation resulting in more widespread activation than voluntary contraction. (g–j) timeline of the BOLD-signal (average over 5 stimulus periods) in the respective clusters marked with circles in map (d). Red, painful condition, blue, non-painful condition, black bar, stimulus condition in event-related block design; error bars represent S.E.M. (g) contraction of the left thigh (control) and contraction of the right painful thigh both lead to an increase in BOLD signal in the right hemisphere in areas of the pre- and postcentral gyrus (blue timeline: control; red timeline: painful contraction of right-sided quadriceps); (h) painful contraction of the right thigh (red timeline) reveals a large increase in BOLD signal in somatotopically related areas of the left pre- and postcentral gyrus (red), similarly, the control contraction leads to coactivation in the left hemisphere (blue timeline); (i) BOLD signal in response to left-sided and right-sided physical stimulation (blue timeline and red timeline); (j) right-sided painful physical stimulation (red timeline), but not left-sided physical stimulation (blue timeline), leads to an increase in BOLD signal in the left-sided somatotopically related areas. Note: panels d-f were magnified by 40% in comparison to a–c.

As expected, the vast majority of regions were activated by painful contraction or physical stimulation of the right quadriceps muscle group, while non-painful contraction or physical stimulation of the left thigh led primarily to activation in the respective somatotopically related areas of the primary sensory and motor area of the contralateral (right) cortex **(** **Fig.** **3** **,** **4** **)**. All figures display significant focal activation with at least t>4.5.

**Figure 4:**
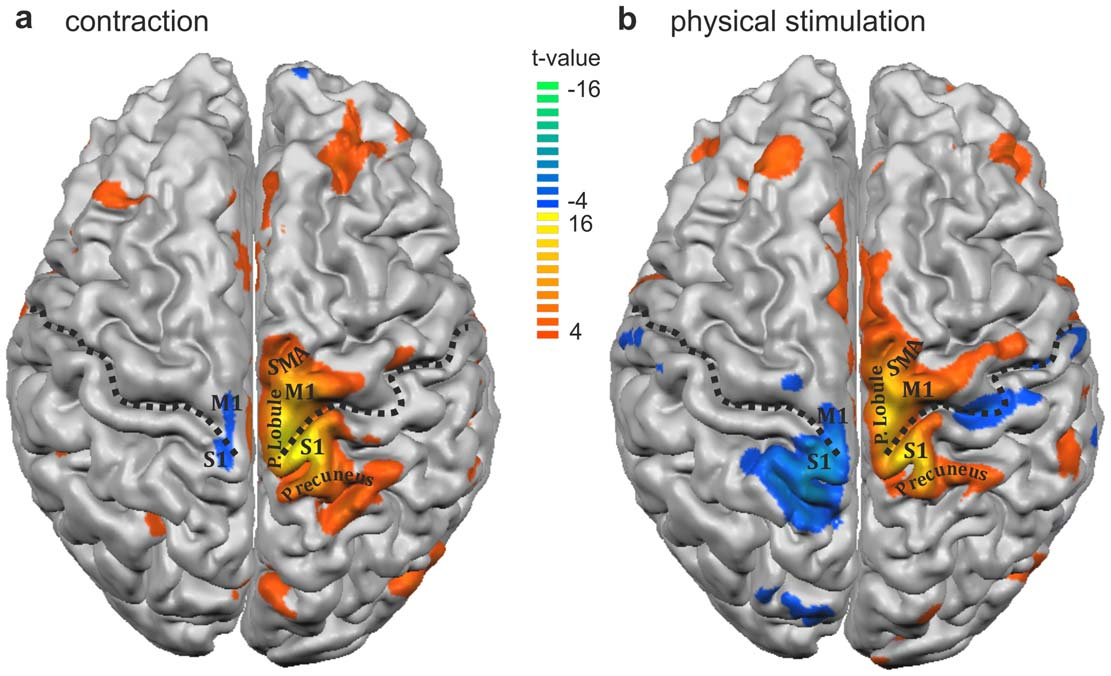
Three-dimensional projection of DOMS-related activation clusters onto the cortical surface. The result of the multi study analysis is projected onto the surface reconstruction of a single brain after Tailarach transformation in response comparing **(a)** contraction and **(b)** physical stimulation**.** Dotted lines represent the central sulcus; SMA: supplementary motor area, M1: primary motor area, S1: primary sensory area; P Lobule: paracentral lobule; Color code: blue-green clusters: higher changes in BOLD-signal during **(a)** non-painful contraction or **(b)** non-painful physical stimulation of the left-sided thigh, red-yellow: higher changes in BOLD-signal during **(a)** painful contraction or **(b)** painful physical stimulation of the right-sided painful thigh; strong activation was located bilaterally in the cingulate motor area.

### Activation in the frontal, parietal and temporal lobe

In general, cortical activation induced by painful physical stimulation of the sore muscle appeared larger, but with respect to its location almost entirely congruent with the contraction-induced activation. During both painful muscle contraction and painful physical stimulation we found the most powerful activation to be located in the contralateral, left-sided primary motor and sensory areas: large clusters were located in the left postcentral gyrus at the vertex, corresponding to the projection area of the primary sensor area (S1) and somatotopically related to the right lower extremity. Furthermore the activation expanded over the cingulate sulcus and merged with activation in the frontal lobe, the latter ones spanning the medial aspect of the fronto-parietal junction, i.e. the paracentral lobule, up to the medial frontal gyrus **(** **Fig.** **3** **,** **4** **)**. The activation appeared as one large cluster not confined to the respective somatotopic area of the thigh but covering also the areas which are thought to correspond to the trunk and the lower limb. This is illustrated in **Fig.** **4** **,** which shows a three-dimensional reconstruction of the activation pattern during DOMS-related pain projected onto the cortical surface for both means of stimulation, contraction **(** **Fig.** **4a** **)** and physical stimulation **(** **Fig.** **4b** **)**. The more rostral located frontal lobe activation corresponds to the medial frontal gyrus which is the projection area of the supplementary motor area (SMA, **Fig.** **3** **,** **4**). In these three areas, S1, M1 and SMA, pain-related t-values were by far the highest for both means of stimulation (compare table 1).

Contraction of the left sound quadriceps muscle activated a small cluster located at the vertex of the contralateral hemisphere spanning the fronto-parietal junction with its centre located in the postcentral gyrus (**Fig.** **3** **,** **4a**). This area corresponds to the somatotopic representation of the left thigh. As expected, painless physical stimulation with the rolling pin led to a more extensive activation of S1 than M1, as represented in **Fig.** **3d–f** **,** **4b** **.** Surprisingly, the cortical surface area of S1 activated by painful contraction or painful physical stimulation was comparable to the area activated by non-painful physical stimulation **(** **Fig.** **4** **)**. This was the contrary for M1, where non-painful means of stimulation only activated a very small area (compare **Table** **1**).

In the left hemispheres the described clusters terminated for both, contraction and physical stimulation, more posterior in the medial parietal lobe with the centre located in the anterior precuneus, which represents the portion of the parietal lobe located caudal to the marginal sulcus and rostral to the parietooccipital sulcus **(** **Fig.** **3** **,** **4** **)**.

We also found an increased pain-related BOLD-signal in the inferior parietal lobule for both contraction and physical stimulation (IPL, see **Table** **1**), Similarly, for both means of stimulation, an increase in pain-related BOLD-signal was noted in the superior temporal gyrus (STG) posterior to the primary auditory cortex in the left hemisphere (compare **Table** **1**).

### Activation in the insula

**Figure 5:**
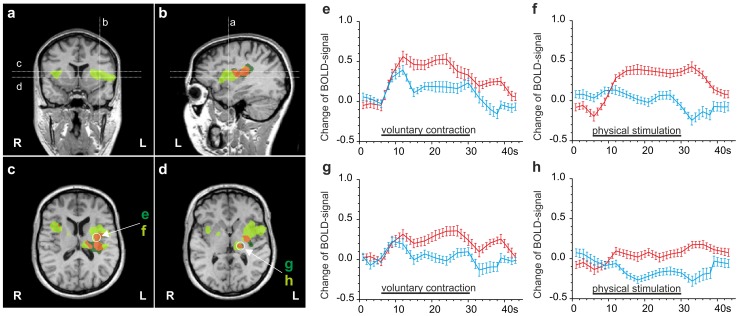
Comparison of DOMS-related activation by contraction and physical stimulation in the insular cortex and ventral posteromedial thalamic nucleus (VPM). Multi studies of activation following both means of stimulation in a standardized Talairach brain. Clusters were calculated by GLM contrasts between painful and non-painful stimulation conditions (t≥5). Dark green: activation by painful contraction; light green: activation by painful physical stimulation; orange: intersection. **a, b.** coronal and sagittal section illustrating orientation of transversal sections in c and d. **a, b, c, d.** Physical stimulation resulted in much larger areas of activation bilaterally in the AIC (a, c) and the left VPM (d), while activation in the MIC and PIC largely overlapped with areas activated during the contraction-related task (b, c). **e, f.** timeline of the BOLD-signal (average over 5 stimulus periods) for painful contraction (red) versus physical stimulation (f) in the same area of the left MIC (circled cluster in c). Painless contraction, but not painless physical stimulation) leads to coactivation during painless contraction (blue) as seen in **Fig. 3**; **g, h.** timeline of the BOLD-signal (average over 5 stimulus periods) for painful contraction versus painful physical stimulation (red timelines) (f) in the same area of the left VPM (circled cluster in d). Again, painless contraction, but not physical stimulation leads to coactivation (blue timelines).

In the insular cortex, we found extensive left-sided activation with painful physical stimulation spanning from the anterior to the posterior part, whereas activation by painful contraction covered a much smaller area limited to the mid- and posterior insular cortex (**Fig.** **5** **;** t-values for activation in the anterior insula remained <5). Furthermore, in contrast to painful contraction, painful physical stimulation led to a large cluster of activation in the right-sided antero-medial insula.

### Activation in the cingulate cortex

**Figure 6:**
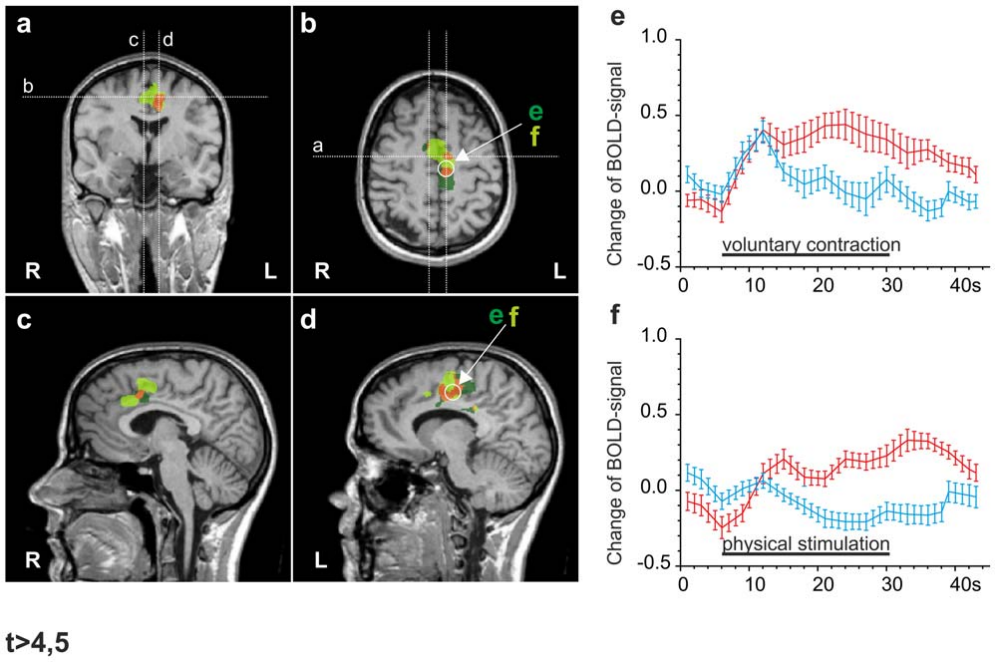
Comparison of DOMS-related activation by contraction and physical stimulation in the cingulate cortex. Multi studies of activation following both means of stimulation in a standardized Talairach brain. Clusters were calculated by GLM contrasts between painful and non-painful stimulation conditions (t≥4.5). Dark green: activation by painful contraction; light green: activation by painful physical stimulation; orange: common activation by both painful stimuli. **a, b.** coronal and transverse section showing orientation of sagittal sections in c and d. **c.** Painful physical stimulation activates more widespread areas of the right posterior ACC. **d.** In the CMA contraction and physical stimulation activate similar areas, albeit physical stimulation-activated areas are more anterior. **e, f.** timeline of the BOLD-signal (average over 5 stimulus periods) for painful contraction (e) and physical stimulation (f) shown as red timelines in the same cluster of the CMA (circled cluster in b and d) compared to the respective painless contraction/stimulation (blue timeline). Painless contraction, but not physical stimulation, leads to coactivation of the contralateral cortical area.

We found activation located in the right posterior anterior cingulate cortex (posterior ACC), which was activated more extensively by physical stimulation than by contraction **(** **Fig.** **6** **,** **Table** **1** **).** A second cluster of activation was located in the left-sided mid-cingulate cortex (MCC, **Fig.** **6a** and **Table** **1**), an area implicated in motor processing and also named the cingulate motor area, CMA. This area was activated during both physical stimulation and contraction. In the posterior cingulate cortex (PCC) we found bilateral clusters of activation, albeit stronger in the right hemisphere.

### DOMS pain-related BOLD-signals in subcortical areas: thalamus and cerebellum

Subcortical activations were confined to the thalamus, basal ganglia and the cerebellum (see **Table** **1**). Painful contraction produced two unilateral clusters of activation in the thalamus. One was located on the left side in the ventral nuclei and the second one in the right-sided medial dorsal part (see **Table** **1**). Painful physical stimulation induced unilateral left-sided activation located close to the area of the ventral posterior nucleus. In contrast to contraction, physical stimulation also activated the right-sided lentiform nucleus **(** **Fig.** **5** **)**.

**Figure 7:**
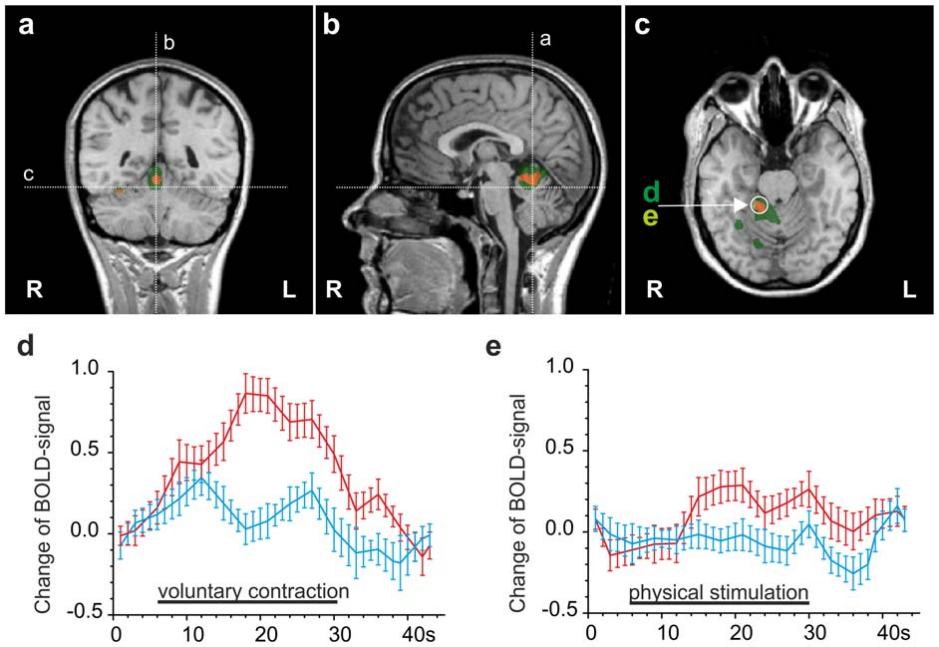
Comparison of DOMS-related activation by contraction and physical stimulation in the cerebellum. Multi studies of activation following both means of stimulation in a standardized Talairach brain. Clusters were calculated by GLM contrasts between painful and non-painful stimulation conditions (t≥5). Dark green: activation by painful contraction; light green: activation by painful physical stimulation; orange: common activation by both painful stimuli. **a, b, d.** coronal and sagittal section showing orientation of transverse sections in c. **c.** In the cerebellum, contraction activates widespread areas. **d, e.** timeline of the BOLD-signal (average over 5 stimulus periods) for painful contraction (d) and painful stimulation (e) in the same cluster of the anterior cerebellum (circled cluster in c) shown as red timelines. Painless contraction/ painless physical stimulation shown as blue timeline.

Activation in the cerebellum was confined to the right brain, ipsilateral to the painful stimulation. Remarkably, in the cerebellum painful contraction produced much larger clusters of activation, also expanding to the posterior lobe, while physical stimulation resulted only in a small cluster located in the anterior lobe **(** **Fig.** **7** **,** **Table** **1** **).**

## Discussion

### DOMS-related pain results in widespread activation in the contralateral primary motor and sensory cortex

In the present study, we describe widespread DOMS-related BOLD-signal increase in S1, M1 and the adjacent areas of the SMA, an area involved in the planning and execution of voluntary movements.

The primary and secondary somatosensory cortex (S1, S2) are amongst those brain regions that consistently respond to painful stimulation with an increase in BOLD signal. In contrast to cutaneous pain, muscle pain leads to signal intensity increase in both the primary sensory and motor cortex (M1) as demonstrated previously using intramuscular electrical stimulation (IMES) and hypertonic saline [13], [28]. In addition muscle pain results in a more widespread signal increase in the primary somatosensory (S1) cortex than cutaneous pain which has been attributed to the fact, that muscle pain is perceived as less well localized than cutaneous pain [29].

Based on Penfield's somatotopic organization of S1 and M1 we expected activation primarily in the region around the vertex, which comprises those portions of the precentral and postcentral gyri that process sensorimotor signals related to the thigh. This was the case for painless contraction. Painful contraction and painful physical stimulation lead to more widespread activation of M1 and S1, extending to areas which are normally activated during sensory and motor stimulation of the upper and lower limb and the trunk. This widespread activation in presence of DOMS-related pain could underlie a transient reorganization of the somatosensory and motor cortex and be directly related to the painful experience, because activations in these areas are even larger during the more painful physical stimulation.

DOMS is a subacute condition and usually entails pain for 24–72 hours that betimes results in maintenance of a protective posture. Previous studies dealing with chronic pain, such as the complex regional pain syndrome (CRPS), phantom limb pain and musculoskeletal pain conditions, like back pain, describe alterations in the somatotopic organization of the primary somatosensory cortex (S1). The amount of this reorganization was correlated with the subjective pain rating and these changes, which are based on processes of neuronal plasticity, could partially be reversed by analgesic interventions in back pain or progressive healing in CRPS [30]–[32]. This widespread activation could also be caused by the fact that pain and soreness were present in a very large area. The quadriceps muscle comprises four muscles, the biarticular rectus femoris, and the three monoarticular vasti muscles (vastus medialis, vastus lateralis, vastus intermedius); in addition, pain was also present in the tensor fasciae latae and the insertion points of the iliotibial tract. It is likely that this widespread activation not only disserves stimulus localization, but also relates to pain intensity. The first aspect is corroborated by lesion studies where destruction of the somatosensory cortex resulted in a greatly impaired stimulus localization without altering the pain affect [33]. In addition, the activation in M1 and SMA were even larger during physical stimulation of the painful limb, the stimulus condition which was also rated as more painful.

Apart from that, we found activation in the precuneus, an area which belongs to the parietal association cortex. Here, activation may result from the inherent task, which required the recognition of the respective light signal (green or red) and its implementation into the appropriate motor action. Similarly, during physical stimulation the subjects could see the red and green light and anticipate the sequence of physical stimulation induced by the experimenter.

### DOMS-related pain leads to activation in the anterior and posterior cingulate cortex and the cingulate motor area

Many previous pain studies (using both PET and fMRI) identified increases in regional cerebral blood flow (rCBF) or BOLD-signal in the cingulate cortex [13], [22], [34]–[36]. In response to DOMS-induced pain, we found activation in the right-sided posterior anterior, the left medial and, bilaterally in the posterior cingulate cortex (post. ACC, MCC and PCC, respectively).

While the ACC is implicated in the processing of affective and reactive components of pain [37] it is also activated by various attention and motor tasks not related to pain [38]. Tonic muscle pain (induced by high-frequency IMES) and cutaneous pain (induced by phasic laser stimuli) resulted in activation of the same areas in the ACC (Brodmann Area 24) [39] as we identified for DOMS. Other studies involving muscle pain in combination with PET also reported activation of the ACC (BA32) [16].

The cingulate motor area (CMA), forming part of the MCC, has previously been implicated in the processing of sensory stimulation, including pain of muscular origin [16]. The CMA was also identified by a group of authors as an area of the cingulate cortex that responds to muscle but not to cutaneous pain [28], [40]. In addition tonic muscle pain was shown to activate the MCC more effectively than phasic muscle pain [29].

The bilateral activation seen in the PCC may be related to pain, which is implied by its activation in response to both stimulation paradigms, but may also relate to the effort of intentionally inducing pain by active muscle contraction. The PCC maintains extensive parietal lobe connections and is thought to be involved in visuospatial orientation and assessment of self-relevant sensation [41].

### DOMS-related pain leads to bilateral activation of the insular cortex

In our study, DOMS-related pain evoked by self-controlled contraction led to large activation in the mid-posterior and posterior insula of the left brain, i.e. contralateral to the painful stimulus and a small area of activation in the right-sided anterior insula. In contrast DOMS pain evoked by physical stimulation led to activation overlapping with these areas including large clusters of activation bilaterally in the anterior insula. This is in agreement with previously reported findings that demonstrate the mid-posterior insula as constantly activated during tonic muscle pain [29]. The bilateral anterior insula is consistently activated with cutaneously applied heat-pain [42], [43], muscle pain applied via deep injection of hypertonic saline [40], IMES [13] and visceral pain, such as esophageal distension [43]. Here, DOMS-related pain only activated the bilateral anterior insula when evoked by the more painful physical stimulation.

### DOMS-pain related activation in the superior temporal gyrus (STG) and the inferior parietal lobule (IPL)

Classically the STG (BA41, 42 and 22) represents the primary auditory receptive cortex and auditory association areas. Further research indicates that the adjacent sulcus (located below BA22 and rostral of BA39), the superior temporal sulcus (STS), is activated by the perception or observation of biologically relevant movements or by the perception of implied movements [44]. The STS is reciprocally connected with the amygdala [44] which loops back to the prefrontal and the motor cortex, including the basal ganglia, completing a pathway from perception to action [45]. Thus the STS and STG might represent a link between the processing of movement, perception and pain. In our experiment, the STG was activated in response to both stimulation paradigms. Previously increases in regional cerebral blood flow (rCBF) or BOLD signal have been described using IMES or intramuscular injections of hypertonic saline [15], [17].

The inferior parietal lobule is an area that represents a boundary between secondary somatosensory areas (S2) and the parietal association cortex and is an area of sensory processing and sensorimotor integration [46]. Previous studies identified activation using IMES [13], [39], [47]. The IPL is adjacent to the secondary somatosensory area (S2) and it was suggested that activation of the IPL (together with S1, S2 and the insula) subserves sensory-discriminative components of pain including encoding of stimulus properties (intensity, localization, duration, and spatial and temporal discrimination)[29]. We found activation in the IPL using both stimulation paradigms.

### Painful and non-painful voluntary movements underlie a disperse processing pattern

Previous studies related to voluntary movements showed that unilateral movements result in a bilateral activation of M1 in the respective homotopic representations arguing for a principle of distributed processing in M1 [48]. We observed this coactivation not only in areas of the supraspinal motor system, including the cerebellum, but also in the insula and the cingulate cortex. BOLD signal intensities increased bilaterally in response to the painful stimulus, but correlations to the applied hemodynamic reference function were highest in M1. We propose that specific unilateral painful (and to a lesser extent non-painful) voluntary movements are accompanied by a global activation of the respective (motor, sensory and sublobar) areas, reflecting an overall increase in neuronal activity and pointing towards a more disperse processing pattern of painful motor movements. This is corroborated by the finding that involultary painful physical stimulation of the same painful muscle did not lead to comparable increases in the BOLD-signal although the pain percieved was rated to be more intense.

### The thalamus and the cerebellum are subcortical areas activated by DOMS-related pain

According to previous findings, the ventral posterior complex in the thalamus is undoubtedly relevant for pain and temperature transmission in humans [49], [50]. In contrast, several studies using PET and fMRI failed to identify activity in the thalamus in response to tonic muscle pain [15], [16], [28], but not to phasic pain [51]. We found a more pronounced and widespread increase in the BOLD-signal in the thalamus in response to physical stimulation and thus the signal-increase in the thalamus may also be related to the pain intensity.

Concerning the cerebellum, pain-specific event-related imaging studies showed that certain cerebellar areas (vermis and lateral posterior hemispheres) seem to be involved in the processing of pain and the nociceptive leg withdrawal reflex [52]. We found activation in the posterior lobe only during the contraction-related task. And, in general, activation in the cerebellum was more widespread during DOMS-related pain evoked by voluntary movement, than by physical stimulation devoid of limb movement. In view of the function of the cerebellum in the programming of voluntary limb movements this is not surprising, but probably ipsilateral cerebellar activation is stronger and more dispersed when voluntary movements are handicapped by nociceptive afferent input incurring from the moving limb. The rather small cerebellar activation in response to passive physical stimulation supports this notion.

## Conclusions

Using event-related fMRI, the temporal evolution of brain activity during the subacute pain state DOMS was mapped in the human brain. We located strongest and widespread pain-related activations in the primary motor and sensory cortex that affected the area somatotopically related to the thigh and also adjacent areas reminiscent of the transient cortical remodelling described in chronic pain states, such as CRPS or phantom limb pain. Further pain-related activation was located in the SMA, IPL, STG, bilaterally in the insula and the cingulate cortex. Activation in the cerebellum was most widespread when pain from DOMS occurred in combination with limb movement. Our study demonstrated that defined stimulation or repeated contraction of a DOMS-affected painful muscle can evoke strong and reproducible increases in BOLD signal; therefore pain from DOMS provides an effective, non-invasive model to study the central processing of inflammatory muscle pain.
